# Impact of COVID-19 on cancer screening in South Korea

**DOI:** 10.1038/s41598-022-15778-3

**Published:** 2022-07-05

**Authors:** Kyeonmin Lee, Yun Yeong Lee, Mina Suh, Jae Kwan Jun, Bomi Park, Yeol Kim, Kui Son Choi

**Affiliations:** 1 National Cancer Center, Graduate School of Cancer Science and Policy, 323, Ilsan-ro, Ilsandong-gu, Goyang, Gyeonggi-do 10408 Korea; 2 National Cancer Center, National Cancer Control Institute, Goyang, Gyeonggi-do Korea; 3 Department of Preventive Medicine, College of Medicine, Chung-Ang University, Seoul, Korea

**Keywords:** Cancer prevention, Cancer screening

## Abstract

The coronavirus disease (COVID-19) pandemic significantly declined cancer screening rates worldwide. Its impact on the South Korean population is unclear, depending on socioeconomic status (SES), residence, and history of chronic disease. This study utilized data (2018–2020) from the Korean National Cancer Screening Survey, an annual cross-sectional study employing nationally representative random sampling. Cancer screening rates were defined as the proportion of the eligible population who received respective cancer screening within the last 1 year and investigated four major cancers (stomach, colorectal, breast, and cervical). Screening rates every year were compared with screening rate ratios (SRRs) and the corresponding 95% confidence intervals (CIs). Between 2019 and 2020, screening rates declined significantly by 23%, 17%, 12%, and 8% for colorectal cancer (SRR 0.77; 95% CI 0.73–0.82), stomach cancer (SRR 0.83; 95% CI 0.79–0.87), breast cancer (SRR 0.88; 95% CI 0.82–0.93), and cervical cancer (SRR 0.92; 95% CI 0.87–0.97), respectively. Regardless of cancer type, screening was significantly lower in metropolitan residents, those with higher SES, and, interestingly, those without a history of chronic diseases. The significant decline in cancer screening during the pandemic requires urgent political intervention to reduce the burden of future cancer incidence and mortality.

## Introduction

At the end of 2019, severe acute respiratory syndrome coronavirus 2 (SARS-CoV-2) emerged, and the World Health Organization declared a global pandemic of the disease, named the coronavirus disease 2019 (COVID-19) on March 11, 2020^1^. Besides its social and economic impact due to quarantine protocols and enforcement of stay-at-home guidelines to reduce disease transmission, cancer control activities worldwide have been significantly affected.

Cancer screening aims to cure cancer by detecting the malignancy or its precursor lesion at an early stage before the onset of symptoms when treatment is most effective^2^. Therefore, the benefits of cancer screening programs are dependent on high coverage of the eligible population as well as complete, timely, and accurate diagnostic follow-up^3^. However, multiple studies have reported that since the emergence of COVID-19, there have been considerable reductions in cancer screening, diagnostic services, endoscopic procedures, and screening-related tests such as biopsies and cervical cytology^3–8^. Similarly, in South Korea, the overall national cancer screening rate was reduced by 6.4% (55.6% in 2019 and 49.2% in 2020)^9^ and breast cancer diagnoses were reduced by 9.9% in 2020^10^.

Routine screening has been postponed largely because of the fear of contracting COVID-19 at a healthcare institution. Nevertheless, delays in cancer screening are estimated to lead to adverse effects regarding future cancer mortality. According to a modeling study conducted in the United Kingdom, delays in diagnosis and treatment due to COVID-19 may increase mortality from breast, colorectal, and lung cancers by up to 9.6, 16.6, and 5.3%, respectively, after 5 years^11^. Therefore, it is important to analyze the impact of COVID-19 on cancer screening, and there is an urgent need for policy interventions to encourage screening. However, such evidence is largely unavailable in Korea. In addition, particular precautions were directed toward high-risk populations, including older adults and those with chronic health conditions, who are more likely to develop severe complications from COVID-19^12^. Moreover, several studies reported that COVID-19 had a greater negative association with cancer screening according to socioeconomic status (SES), geographical region, race, and ethnicity than before the COVID-19 pandemic^13–15^. This may indicate that pre-existing disparities in cancer screening may be exacerbated by COVID-19, with long-term consequences affecting cancer incidence and mortality. Therefore, the goal of this study was to (i) measure the impact of COVID-19 on cancer screening by comparing screening rates during the pre-and post-COVID-19 eras, between 2018 and 2020, and (ii) investigate the associations of COVID-19 with cancer screening according to SES, residential regions, and a history of chronic diseases.

## Methods

### Study population

This study used data from the Korean National Cancer Screening Survey (KNCSS) from 2018 to 2020. The KNCSS is an annual, nationwide, population-based study that has been conducted since 2004 to determine the current status of both organized and opportunistic cancer screening rates for five major cancer types, including stomach, liver, colorectal, breast, and cervical cancer^16^. To obtain a nationally representative sample, survey participants were selected by stratified, multi-stage random sampling based on geographical area, age, and sex^16^. Following door-to-door recruitment of participants, data were collected through face-to-face interviews by a professional research agency. All study participants agreed to participate in the survey after obtaining adequate information regarding the study^17^.

The survey involved cancer-free men and women aged > 40 years and > 30 years, respectively, based on the National Cancer Screening Program protocols in Korea^18^. In 2015, the national cervical cancer screening program was extended to include women in their 20 s^17^. Hence, these women were only included in the calculation of the cervical cancer screening rates. A total of 4500 men and women completed the survey in 2018, 2019, and 2020 and were included in the final analysis. The requirement for written informed consent was waived by the institutional review board (IRB) of the National Cancer Center, Korea because the proposed research presents no more than a minimal risk of harm to subjects. This study was approved by the IRB (Approval Number: NCC-2019-0233). All methods were carried out in accordance with approved guidelines and regulations.

### Measures

**Figure 1:**
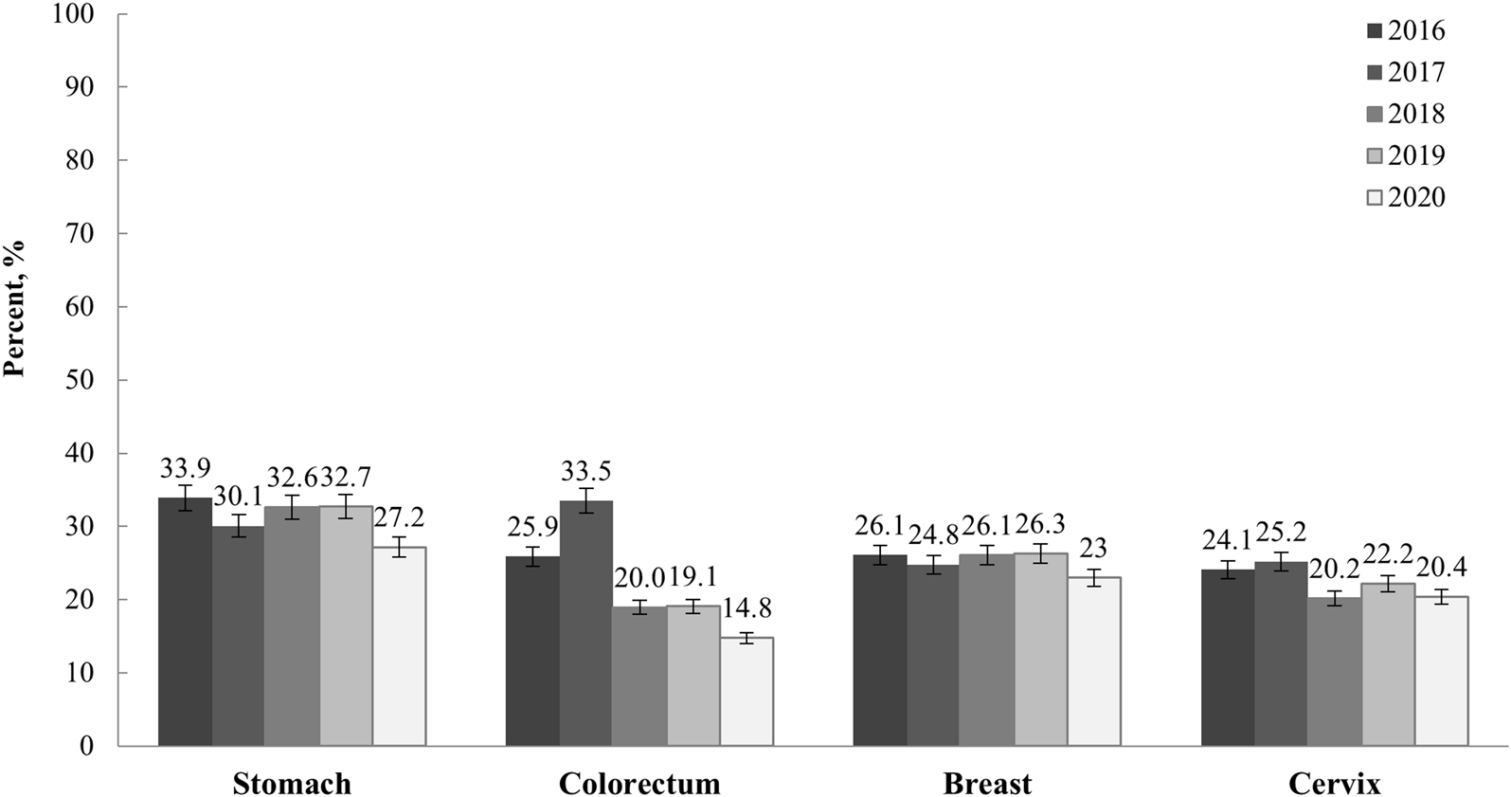
Latest cancer screening rates within the last 1 year for the period 2016–2020. The cancer screening rates were measured according to cancer screening protocols issued by the NCSP of Korea: Stomach, Percentage of adults aged $\ge$ 40 years who underwent stomach cancer screening using either UGIS or endoscopy. Colorectum, Percentage of adults aged $\ge$ 50 years who underwent colorectal cancer screening using FIT. Breast, Percentage of women aged $\ge$ 40 years who underwent breast cancer screening using mammography. Cervix, Percentage of women aged $\ge$ 20 years who underwent cervical cancer screening using conventional cytology. Weighting values from the Population and Housing Census were applied to calculate the screening rates. UGIS, gastrointestinal series; FIT, fecal immunochemical test.

Using a structured questionnaire, the KNCSS explored the study participants’ experiences with screening for five major cancer types (stomach, liver, colorectal, breast, and cervix), and socioeconomic characteristics including residential area, household income, educational status, and history of chronic disease were recorded. Residential regions were defined into three categories: metropolitan, urban, and rural regions, based on provincial levels in Korea. Amongst 17 administrative districts, 1 capital city and 6 metropolitan cities (Seoul, Busan, Daegu, Incheon, Gwangju, Daejeon, and Woolsan) were categorized as metropolitan. For urban and rural regions, 10 provincial cities (Sejong, Gyeonggi, Gangwon, Chungbuk, Chungnam, Jeongbuk, Jeonnam, Gyeongbuk, Gyeongnam, and Jeju) were categorized as urban, and if the provincial cities have sub-municipal level divisions, either Eup (town) or Myeon (township), they were categorized as rural. We also defined chronic health conditions that may cause severe complications from COVID-19, such as liver diseases, tuberculosis, diabetes, and heart conditions, or those health conditions that require lifetime management because of a high risk of developing cancers^12^. To measure the interviewees’ cancer screening experiences for each cancer type, the questions included “Have you undergone [cancer type] screening?” If so, “When did you last undergo [cancer type] screening?” To investigate the changes in the screening rate due to COVID-19, we estimated the cancer screening rate as having undergone cancer screening within the last 1 year for stomach, colorectal, breast, and cervical cancers based on the cancer screening protocols issued by the NCSP (Fig. 1). We then compared the cancer screening rates of 2019 versus 2018 and 2020 versus 2019 and estimated the screening rate ratios (SRRs) and corresponding 95% confidence intervals (CIs).

### Statistical analysis

The general characteristics of the survey respondents are presented as unweighted numbers and proportions in Supplementary Table S1. To calculate cancer screening rates, we applied survey sample weights from the Population and Housing Census of Statistics Korea. To provide 95% CIs for the SRRs, the standard error (SE) of log SRR was determined using a delta method-derived formula^19^: SE[log(SRR)] = [(p1)/(p1 × n1) + (p2)/(p2 × n2)]^0.5^, where p is the proportion of screened individuals and n is the number of eligible individuals. For example, for the SRR between 2018 and 2019, p1 is the proportion of individuals screened in 2019, n1 is the number of eligible individuals in 2019, p2 is the proportion of individuals screened in 2018, and n2 is the number of eligible individuals in 2018. Therefore, 95% CIs were calculated from: exp[ln(SRR) ± 1.96 × SE]^19^. Statistical analysis was performed using the SAS software (version 9.4; SAS Institute, Inc., Cary, NC, USA).

### Ethical statement

Our study protocol was approved by the National Cancer Center Institutional Review Board of Korea (Approval Number: NCC2019-0233). All participants consented to participate in the survey for public benefit; the requirement for written informed consent was waived.

## Results

The demographic characteristics of the study participants for each year, from 2018 to 2020, are presented in Supplementary Table S1. Demographic distributions by the SES changed with the transition in demographic status for eligible Korean adults. Figure 1 shows the most recent screening rates within the last year, for the period 2016 to 2020. For all four cancers, the cancer screening rates within the last year have been flattened until 2019 and then significantly declined in 2020 compared to those in 2018 and 2019.

Tables 1, 2, 3 and 4 present the screening rates for the previous year and compare the SRRs between 2018 and 2020. Between 2018 and 2019, there were no significant changes in the overall SRR for stomach, colorectal, and breast cancer screening rates, except that the cervical cancer screening rate significantly increased by 10% (SRR 1.10; 95% CI 1.04–1.16). In contrast, the SRRs between 2019 and 2020 markedly decreased by 17% for stomach cancer (SRR 0.83; 95% CI 0.79–0.87; Table 1), 23% for colorectal cancer (SRR 0.77; 95% CI 0.73–0.82; Table 2), 12% for breast cancer (SRR 0.88; 95% CI 0.82–0.93; Table 3), and 8% for cervical cancer (SRR 0.92; 95% CI 0.87–0.97; Table 4).

**Table 1:** Stomach cancer screening rates for the period 2018–2020.

Characteristics	2018 (n = 3495^a^)	2019 (n = 3539^a^)	2020 (n = 3557^a^)	2019 versus 2018			2020 versus 2019		
	SR, %^b^	SR, %^b^	SR, %^b^	SE	SRR	95% CI	SE	SRR	95% CI
Overall	32.6	32.7	27.2	0.024	1.00	0.96–1.05	0.024	0.83	0.79–0.87
**Age group, years**									
40–49	33.8	34.3	26.7	0.041	1.01	0.94–1.10	0.043	0.78	0.72–0.85
50–59	32.6	32.7	28.3	0.041	1.00	0.92–1.09	0.042	0.86	0.80–0.94
60–69	32.7	35.5	27.9	0.050	1.09	0.98–1.20	0.049	0.79	0.71–0.86
70–74	27.0	24.4	24.4	0.077	0.91	0.78–1.05	0.065	1.00	0.88–1.14
**Sex**									
Male	33.8	32.4	26.8	0.034	0.96	0.90–1.02	0.034	0.83	0.77–0.88
Female	31.5	33.1	27.6	0.034	1.05	0.98–1.12	0.033	0.83	0.78–0.89
**Residential area**									
Metropolitan	34.8	33.1	23.2	0.036	0.95	0.89–1.02	0.036	0.70	0.65–0.75
Urban	32.1	34.9	30.3	0.036	1.09	1.01–1.17	0.035	0.87	0.81–0.93
Rural	27.0	22.5	30.1	0.070	0.83	0.73–0.95	0.074	1.34	1.16–1.55
**Monthly household income ($)**									
≤ 3999	28.9	29.6	27.1	0.040	1.03	0.95–1.11	0.040	0.91	0.85–0.99
4000–6999	34.5	30.9	25.1	0.039	0.90	0.83–0.97	0.040	0.81	0.75–0.88
≥ 7000	35.0	39.1	29.4	0.046	1.12	1.02–1.22	0.044	0.75	0.69–0.82
**Education status**									
Middle school or lower	23.1	24.6	26.1	0.062	1.07	0.94–1.20	0.060	1.06	0.94–1.20
High school	34.0	32.1	26.7	0.033	0.95	0.89–1.01	0.033	0.83	0.78–0.89
College or higher	34.5	38.0	28.4	0.042	1.10	1.02–1.20	0.042	0.75	0.69–0.81
**Chronic disease**^**c**^									
No	31.5	33.4	25.7	0.031	1.06	1.00–1.13	0.031	0.77	0.72–0.82
Yes	34.3	31.8	29.1	0.037	0.93	0.86–1.00	0.036	0.91	0.85–0.98

*SR* screening rate; *SRR* screening rate ratio; *SE* standard error; *CI* confidence interval.

^a^The n value is the number of individuals eligible for gastric cancer screening (aged over 40 years); ^b^SRs were defined as the proportion of screened individuals within the last 1 year; Weighting values from the Population and Housing Census were applied to calculate the SRs ^**c**^Chronic disease, individuals who are diagnosed with any of following diseases: hypertension, diabetes, tuberculosis, hepatitis B, hepatitis C, liver cirrhosis, chronic gastritis, stomach/duodenal ulcers, polyps, benign breast diseases, uterine myoma, and hyperlipidemia.

**Table 2:** Colorectal cancer screening rates for the period 2018–2020.

Characteristics	2018 (n = 2277^a^)	2019 (n = 2425^a^)	2020 (n = 2467^a^)	2019 versus 2018			2020 versus 2019		
	SR, %^b^	SR, %^b^	SR, %^b^	SE	SRR	95% CI	SE	SRR	95% CI
Overall	20.0	19.1	14.8	0.029	0.95	0.90–1.01	0.029	0.77	0.73–0.82
**Age group, years**									
50–59	23.4	19.3	16.1	0.041	0.82	0.76–0.89	0.042	0.83	0.77–0.91
60–69	16.7	20.5	14.0	0.050	1.23	1.11–1.35	0.049	0.68	0.62–0.75
70–74	14.7	16.1	13.0	0.077	1.09	0.94–1.27	0.065	0.81	0.71–0.92
**Sex**									
Male	17.2	19.3	14.9	0.042	1.12	1.03–1.22	0.041	0.77	0.71–0.83
Female	22.8	18.9	14.7	0.041	0.83	0.77–0.90	0.040	0.78	0.72–0.84
**Residential area**									
Metropolitan	21.3	20.1	12.3	0.044	0.94	0.86–1.02	0.043	0.62	0.57–0.67
Urban	20.1	19.3	18.4	0.045	0.96	0.88–1.05	0.043	0.95	0.87–1.03
Rural	15.8	15.0	10.5	0.080	0.95	0.81–1.11	0.083	0.70	0.59–0.82
**Monthly household income ($)**									
≤ 3999	16.5	19.3	13.4	0.044	1.17	1.08–1.28	0.043	0.69	0.64–0.75
4000–6999	22.3	16.7	15.0	0.052	0.75	0.68–0.83	0.053	0.89	0.81–0.99
≥ 7000	23.5	21.6	16.9	0.059	0.92	0.82–1.03	0.056	0.78	0.70–0.87
**Education status**									
Middle school or lower	15.0	15.1	13.5	0.062	1.01	0.89–1.14	0.061	0.89	0.79–1.01
High school	20.1	19.6	14.4	0.038	0.97	0.90–1.05	0.037	0.73	0.68–0.79
College or higher	24.9	22.8	17.6	0.067	0.91	0.80–1.04	0.067	0.77	0.68–0.88
**Chronic disease**^**c**^									
No	20.3	19.4	13.5	0.042	0.96	0.88–1.04	0.042	0.70	0.64–0.75
Yes	19.8	18.8	15.9	0.041	0.95	0.88–1.03	0.039	0.84	0.78–0.91

*SR* screening rate, *SRR* screening rate ratio, *SE* standard error, *CI* confidence interval.

^a^The n value is the number of individuals eligible for colorectal cancer screening (aged over 50 years); ^b^SRs were defined as the proportion of screened individuals within the last 1 year; Weighting values from the Population and Housing Census were applied to calculate the SRs; ^**c**^Chronic disease, individuals who are diagnosed with any of following diseases: hypertension, diabetes, tuberculosis, hepatitis B, hepatitis C, liver cirrhosis, chronic gastritis, stomach/duodenal ulcers, polyps, benign breast diseases, uterine myoma, and hyperlipidemia.

**Table 3:** Breast cancer screening rates for the period 2018–2020.

Characteristics	2018 (n = 1754^a^)	2019 (n = 1795^a^)	2020 (n = 1800^a^)	2019 versus 2018			2020 versus 2019		
	SR, %^b^	SR, %^b^	SR, %^b^	SE	SRR	95% CI	SE	SRR	95% CI
Overall	26.1	26.3	23.0	0.034	1.01	0.94–1.08	0.033	0.88	0.82–0.93
**Age group, years**									
40–49	28.4	28.2	24.1	0.059	0.99	0.88–1.11	0.061	0.85	0.76–0.96
50–59	26.6	28.7	26.1	0.059	1.08	0.96–1.21	0.059	0.91	0.81–1.02
60–69	24.5	24.1	21.7	0.069	0.99	0.86–1.13	0.069	0.90	0.79–1.03
70–74	18.4	20.2	16.4	0.105	1.10	0.89–1.35	0.087	0.81	0.68–0.96
**Residential area**									
Metropolitan	24.4	26.9	19.6	0.050	1.10	1.00–1.22	0.050	0.73	0.66–0.80
Urban	29.4	26.4	27.4	0.051	0.90	0.81–0.99	0.050	1.04	0.94–1.14
Rural	20.2	23.2	19.0	0.100	1.15	0.94–1.40	0.105	0.82	0.67–1.00
**Monthly household income ($)**									
≤ 3999	22.4	24.7	22.3	0.057	1.10	0.99–1.23	0.056	0.90	0.81–1.01
4000–6999	27.4	23.6	20.9	0.056	0.86	0.77–0.96	0.058	0.89	0.79–0.99
≥ 7000	29.1	31.3	25.8	0.063	1.08	0.95–1.22	0.060	0.82	0.73–0.93
**Education status**									
Middle school or lower	15.2	18.6	18.0	0.079	1.22	1.04–1.42	0.076	0.97	0.84–1.12
High school	27.7	28.0	23.0	0.044	1.01	0.93–1.10	0.044	0.82	0.75–0.90
College or higher	29.6	28.8	27.0	0.068	0.97	0.85–1.11	0.069	0.94	0.82–1.07
**Chronic disease**^**c**^									
No	25.3	27.4	22.0	0.043	1.08	0.99–1.18	0.044	0.80	0.74–0.88
Yes	27.2	24.5	24.2	0.054	0.90	0.81–1.00	0.052	0.99	0.89–1.09

*SR* screening rate, *SRR* screening rate ratio, *SE*standard error, *CI* confidence interval.

^a^The n value is the number of women eligible for breast cancer screening (aged over 40 years); ^b^SRs were defined as the proportion of screened women within the last 1 year; Weighting values from the Population and Housing Census were applied to calculate the SRs; ^**c**^Chronic disease, women who are diagnosed with any of the following diseases: hypertension, diabetes, tuberculosis, hepatitis B, hepatitis C, liver cirrhosis, chronic gastritis, stomach/duodenal ulcers, polyps, benign breast diseases, uterine myoma, and hyperlipidemia.

**Table 4:** Cervical cancer screening rates for the period 2018–2020.

Characteristics	2018 (n = 2759^a^)	2019 (n = 2756^a^)	2020 (n = 2743^a^)	2019 versus 2018			2020 versus 2019		
	SR, %^b^	SR, %^b^	SR, %^b^	SE	SRR	95% CI	SE	SRR	95% CI
Overall	20.2	22.2	20.4	0.027	1.10	1.04–1.16	0.027	0.92	0.87–0.97
**Age group, years**									
20–29	9.9	14.0	13.0	0.063	1.41	1.25–1.60	0.063	0.93	0.82–1.05
30–39	20.6	27.1	24.3	0.065	1.32	1.16–1.49	0.067	0.89	0.79–1.02
40–49	26.3	26.6	23.1	0.059	1.01	0.90–1.14	0.061	0.87	0.77–0.98
50–59	24.6	25.9	23.9	0.059	1.05	0.94–1.18	0.059	0.92	0.82–1.04
60–69	19.5	19.8	21.4	0.068	1.02	0.89–1.17	0.069	1.08	0.95–1.24
70–74	12.2	15.6	13.1	0.104	1.28	1.04–1.57	0.087	0.83	0.70–0.99
**Residential area**									
Metropolitan	22.2	21.6	17.9	0.040	0.97	0.90–1.05	0.040	0.83	0.77–0.90
Urban	19.4	24.2	23.4	0.040	1.25	1.15–1.35	0.040	0.97	0.89–1.05
Rural	14.8	14.9	17.6	0.086	1.01	0.85–1.19	0.093	1.18	0.99–1.42
**Monthly household income ($)**									
≤ 3999	17.3	20.9	19.5	0.049	1.21	1.10–1.33	0.049	0.93	0.85–1.03
4000–6999	23.8	21.4	19.9	0.045	0.90	0.83–0.98	0.047	0.93	0.85–1.02
≥ 7000	18.9	24.1	21.5	0.047	1.27	1.16–1.40	0.045	0.89	0.82–0.97
**Education status**									
Middle school or lower	11.5	12.9	15.6	0.079	1.12	0.96–1.31	0.075	1.21	1.05–1.41
High school	22.0	23.2	21.2	0.040	1.05	0.97–1.14	0.040	0.92	0.85–0.99
College or higher	20.3	24.1	20.9	0.042	1.19	1.09–1.29	0.042	0.87	0.80–0.94
**Chronic disease**^**c**^									
No	17.2	22.8	20.1	0.032	1.33	1.25–1.41	0.032	0.88	0.83–0.94
Yes	27.7	20.4	20.8	0.052	0.74	0.67–0.82	0.050	1.02	0.93–1.13

*SR* screening rate, *SRR* screening rate ratio, *SE* standard error, *CI* confidence interval.

^a^The n value is the number of women eligible for cervical cancer screening (aged over 20 years); ^b^SRs were defined as the proportion of screened women within the last 1 year; Weighting values from the Population and Housing Census were applied to calculate the SRs; ^**c**^Chronic disease, women who are diagnosed with any of following diseases: hypertension, diabetes, tuberculosis, hepatitis B, hepatitis C, liver cirrhosis, chronic gastritis, stomach/duodenal ulcers, polyps, benign breast diseases, uterine myoma, and hyperlipidemia.

Although cancer screening rates significantly decreased between 2019 and 2020, the magnitude of the decline varied according to socioeconomic characteristics. Regardless of cancer type, the largest reductions were observed in those who lived in metropolitan areas, with a 30% reduction for stomach cancer (SRR 0.70; 95% CI 0.65–0.75; Table 1), 38% for colorectal cancer (SRR 0.62; 95% CI 0.57–0.67; Table 2), 27% for breast cancer (SRR 0.73; 95% CI 0.66–0.80; Table 3), and 17% for cervical cancer (SRR 0.83; 95% CI 0.77–0.90; Table 4).

For stomach cancer, the second-largest reductions were seen in those who had a high household income (SRR 0.75; 95% CI 0.69–0.82; Table 1) and a high education status (SRR 0.75; 95% CI 0.69–0.81; Table 1). For colorectal cancer, the second largest reduction was observed among those aged 60–69 years (SRR 0.68; 95% CI 0.62–0.75; Table 2). Among women who are eligible for breast and cervical cancer screenings, a significant decline was observed among older women [70–74 years; SRR 0.81; 95% CI 0.68–0.96 for breast cancer (Table 3); SRR 0.83; 95% CI 0.70–0.99 for cervical cancer (Table 4)].

Based on the history of chronic diseases, considerable reductions were found during the pandemic among those without a history of chronic diseases [23% for stomach cancer (SRR 0.77; 95% CI 0.72–0.82; Table 1), 30% for colorectal cancer (SRR 0.70; 95% CI 0.64–0.75; Table 2), 20% for breast cancer (SRR 0.80; 95% CI 0.74–0.88; Table 3), and 12% for cervical cancer (SRR 0.88; 95% CI 0.83–0.94; Table 4)].

## Discussion

In the current study, screening rates within the last year significantly decreased during the COVID-19 pandemic. These results may indicate a negative impact of COVID-19 on health-seeking behaviors. The magnitude of reduction in cancer screening differed according to socioeconomic characteristics, residential status, and a history of chronic diseases. In compliance with previous studies^20–25^, for all four cancer types, there was a pre-existing disparity in screening prior to the COVID-19 pandemic by SES and residential status. In 2018 and 2019, the screening rates were significantly lower for those with the lowest monthly household income, education level, and residency in rural regions. However, similar to a previous study that quantified cancer screening rates associated with the COVID-19 using medical claims data of 60-million population in the United States^13^, the current study showed that COVID-19 had a greater negative association with a higher SES. In particular, the decline in the screening rate was large among those with high income levels. Gastric and colorectal cancer screening exhibited a significant decline in individuals with high educational levels.

In contrast to other previous studies^14,15^, our study results showed that the magnitude of deficits in cancer screening was smaller among those with a low SES than those with a high SES. For possible reasons, we suspect that the Korean NCSP is provided free of charge for medical aid program recipients or national health insurance service beneficiaries of low-income status. Thus, those with a low SES were less likely to be affected by COVID-19 in comparison to other countries.

In addition to this, the cancer screening rate in metropolitan areas, where many COVID-19 cases have been confirmed, has declined significantly. This might be related to regional quarantine policies, such as refraining from going out and the temporary closure of medical facilities in Daegu and Seoul due to the COVID-19 pandemic in February, March, and May 2020. Moreover, those with a high SES are more likely to reside in metropolitan regions. Therefore, it can be understood that individuals with a high SES have significantly reduced the use of cancer screening services than those with a low SES due to a higher likelihood of COVID-19 transmission. Although there were disparities in cancer screening by SES and residential area before the pandemic, this gap narrowed as the screening rate decreased due to COVID-19.

Interestingly, the magnitude of the reduction in cancer screening was significantly greater in individuals without chronic health conditions than in those with chronic health conditions. In other words, during the pandemic, individuals without chronic health conditions tried to refrain from using medical services, including cancer screening, while individuals with chronic health conditions continued to use medical services. These results raise great concerns about the direct impact on cancer screening when asymptomatic individuals underestimate their risk of developing cancer and refrain from visiting screening facilities. According to a previous study conducted in Korea^26^, there was a significant upward trend in the rates of non-participation in pre-scheduled health check-ups when the fear of COVID-19 exceeded that of lung cancer; the prevalence of the fear of COVID-19 was approximately 30% in the general public. Our results suggest that excessive fear of COVID-19 greatly hindered screening for those who perceive themselves as being at low risk of developing cancer.

For female-specific cancers, including breast and cervical cancers, we have noted that older adult women aged 70–74 years experienced greater declines compared to women in other age groups. A previous study reported that women exhibited significantly higher levels of fear of COVID-19 than men, and older adults also had higher risk perceptions of COVID-19^27^. These findings align with those of previous studies showing that women tend to have greater psychological vulnerability or fear of COVID-19^28–30^. However, the factors associated with lower cancer screening participation in older adult women may be multifactorial, and more research will be needed to determine why those women reduced the use of cancer screening services more than younger women.

Although South Korea’s robust public health response to COVID-19 has successfully slowed the spread of the disease without major lockdowns and less stringent national social distancing policies relative to countries such as the US, Italy, France, and the UK^31^, fear of COVID-19 was persistent in the public and it significantly affected cancer prevention activities in Korea. We have noted that declines in cancer screening were much larger for gastric cancer and colorectal cancer, which require endoscopic procedures for screening or diagnostic tests. Unlike other western countries, the Korean NCSP provides gastric cancer screening using endoscopy or alternative UGIS every 2 years due to a high incidence of gastric cancer. In addition, an annual FIT is recommended for colorectal cancer screening, and those who receive positive results from the FIT are further referred to confirmative colonoscopy. We assume that people are more reluctant to receive screening tests that involve endoscopic procedures compared with breast and cervical cancer screening, possibly because of a higher chance of aerosol transmission or fecal shedding of COVID-19. However, further studies will be necessary to ascertain the reasons for the different magnitudes of deficits caused by cancer screening.

Delays in early detection due to the decline in cancer screening may result in severe future consequences, such as an unexpected rise in cancer incidence, a higher proportion of later-stage cancer diagnoses, and, in turn, increased cancer mortality. A population-based study conducted in Canada estimated that 6 months interruptions in breast and colorectal cancer screening could lead to 670 extra cases of advanced stages of breast cancer, and the early detection of 19,000 adenomas and colorectal cancers would be missed^32^. This, in turn, may eventually lead to 250 and 960 extra cancer deaths for breast and colorectal cancer, respectively.

Hence, even during the pandemic, it remains important to improve public awareness of cancer prevention and promote sustainable behavioral changes while strengthening compliance with cancer screening guidelines.

This study has some limitations. Because the survey data were primarily based on the study participants’ self-reports, recall bias could potentially be introduced when describing previous screening experiences or SES. Second, the cross-sectional study design means that long-term consequences of delayed screening were not measurable. Therefore, modeling studies to estimate the additional cancer incidence and mortality caused by deficits in cancer screening due to COVID-19 are required to evaluate its long-term impact. Despite these limitations, to our knowledge, this is the first population-based study to demonstrate that deficits in both opportunistic and organized cancer screening rates differed according to SES, residential region, and history of chronic diseases during the COVID-19 pandemic in South Korea.

We observed a significant decline in cancer screening during the COVID-19 pandemic in South Korea. The greatest reductions in cancer screening were significantly associated with high SES, residency in metropolitan areas, older adult women, and having no chronic health conditions. Delays in cancer screening could lead to adverse consequences such as increased cancer incidence and mortality in the future. Urgent political intervention to increase the uptake of cancer screening should be considered to compensate for the deficits caused by COVID-19.

## Supplementary Information

Supplementary Information.

## Data Availability

The data supporting the findings of this study are available from the corresponding author upon reasonable request.
