# Supplementary material for: Impact of COVID-19 on cancer screening in South Korea
Source: Sci Rep. 2022 Jul 5;12:11380. doi: 10.1038/s41598-022-15778-3 (PMC9255521; doi:10.1038/s41598-022-15778-3)
Supplement: Supplementary file 1 — Supplementary Information. [file 41598_2022_15778_MOESM1_ESM.docx]

| **Supplementary table S1.** Baseline characteristics of study participants | | | |
| --- | --- | --- | --- |
| **Characteristics** | **2018** | **2019** | **2020** |
|  | N, %^a^ | N, %^a^ | N, %^a^ |
| **Total** | 4 500 (100.0) | 4 500 (100.0) | 4 500 (100.0) |
| **Age group**, years |  |  |  |
| 20–29 | 500 (11.1) | 500 (11.1) | 500 (11.1) |
| 30–39 | 505 (11.2) | 461 (10.2) | 443 (9.8) |
| 40–49 | 1 218 (27.1) | 1 114 (24.8) | 1 090 (24.2) |
| 50–59 | 1 204 (26.8) | 1 147 (25.5) | 1 131 (25.1) |
| 60–69 | 814 (18.1) | 808 (18.0) | 855 (19.0) |
| 70–74 | 259 (5.8) | 470 (10.4) | 481 (10.7) |
| **Sex** |  |  |  |
| Male | 1 741 (38.7) | 1 744 (38.8) | 1 757 (39.0) |
| Female | 2 759 (61.3) | 2 756 (61.2) | 2 743 (61.0) |
| **Residential area** |  |  |  |
| Metropolitan | 2 015 (44.8) | 2 001 (44.5) | 2 079 (46.2) |
| Urban | 1 948 (43.3) | 2 057 (45.7) | 2 039 (45.3) |
| Rural | 537 (11.9) | 442 (9.8) | 382 (8.5) |
| **Monthly household income ($)** | |  |  |
| ≤3,999 | 1 494 (33.2) | 1 418 (31.5) | 1 540 (34.2) |
| 4,000–6,999 | 1 703 (37.8) | 1 693 (37.6) | 1 441 (32.0) |
| ≥7,000 | 1 303 (29.0) | 1 389 (30.9) | 1 519 (33.8) |
| **Education status** |  |  |  |
| Middle school or lower | 484 (10.8) | 583 (13.0) | 527 (11.7) |
| High school | 2 096 (46.6) | 2 078 (46.2) | 2 142 (47.6) |
| College or higher | 1 920 (42.7) | 1 839 (40.8) | 1 831 (40.7) |
| **Chronic disease**^b^ |  |  |  |
| No | 3 032 (67.4) | 3 018 (67.1) | 2 874 (63.9) |
| Yes | 1 468 (32.6) | 1 482 (32.9) | 1 626 (36.1) |
| ^a^Frequencies and proportions are not weighted;  ^b^Chronic disease, individuals who are diagnosed with any of following diseases: hypertension, diabetes, tuberculosis, hepatitis B, hepatitis C, liver cirrhosis, chronic gastritis, stomach/duodenal ulcers, polyps, benign breast diseases, uterine myoma, or hyperlipidemia | | | |
